# Artemisinin ameliorates thyroid function and complications in adult male hypothyroid rats via upregulation of the L1 cell adhesion molecule

**DOI:** 10.1186/s13044-024-00206-7

**Published:** 2024-08-19

**Authors:** Lingling Li, Haifan Xu, Zecheng Hu, Li Li

**Affiliations:** 1 Department of Breast and Thyroid Surgery, The First Affiliated Hospital, Hengyang Medical School, University Of SouthChina, Hengyang, Hunan 421001 China; 2 Department of Gastrointestinal Surgery, The First Affiliated Hospital, Hengyang Medical School, University of South China, Hengyang, Hunan 421001 China

**Keywords:** Hypothyroidism, Artemisinin (ART), L1, Oxidative stress

## Abstract

**Background:**

Hypothyroidism, a common worldwide syndrome caused by insufficient thyroid hormone secretion, affects number of people at different ages. Artemisinin (ART), a well-known effective agent in the treatment of malaria, also has anti-oxidative stress functions in various diseases. The L1 cell adhesion molecule exerts multiple protective roles in diseased systems. The aim of the present study was to evaluate the role of ART in adult male hypothyroid rats and the underlying mechanisms.

**Methods:**

The propylthiouracil (PTU) rat model was treated with or without 5 mg/kg ART and with or without L1 short-interfering RNA (siRNA), followed by the experiments to determine the effect of ART on thyroid function, depression and anxiety, cognition impairments, liver, kidney and heart functions, and oxidative stress.

**Results:**

In the current study, it was shown that ART can ameliorate thyroid function, mitigate depression and anxiety symptoms, attenuate cognition impairments, improve liver, kidney and heart functions, and inhibit oxidative stress; however, the effects exerted by ART could not be observed when L1 was silenced by L1 siRNA.

**Conclusion:**

These results indicated that ART can upregulate the L1 cell adhesion molecule to ameliorate thyroid function and the complications in adult male hypothyroid rats, laying the foundation for ART to be a novel strategy for the treatment of hypothyroidism.

## Background

With an increase in metabolic diseases in recent years, thyroid dysfunctions, especially hypothyroidism, represent the most common endocrine disorders, and are associated with an increased risk of comorbidity which impacts ability to work and quality of life worldwide [1]. Hypothyroidism is a prevalent medical condition characterized by a reduction in the metabolic activity of the body due to a decrease in the synthesis and secretion of thyroid hormones or their efficacy [2]. Due to the notable roles of thyroid hormones in normal functioning of the heart, kidneys, liver and brain, these organs are adversely affected by hypothyroidism [3, 4]. Thyroid hormones have a vital impact on the development and normal functioning of the brain throughout life [5]. Adult onset hypothyroidism is associated with depression, anxiety [6] and memory deficits [7, 8] in humans and in animal models [9, 10]. Thyroid hormones are crucial for oxidative metabolism and energy expenditure [11]. The conventional treatment of hypothyroidism has adverse drug events, high treatment costs and compliance issues; the potential anti-oxidative stress strategies associated with heart, kidney, liver and brain dysfunction may be useful for the treatment of hypothyroidism.

Attempts to apply beneficial molecules that may treat hypothyroidism have led to experiments using animal models and have, to some extent, ameliorated the complications. Among several molecules, artemisinin (ART) is a compound first discovered in the 1970s and is derived from sweet wormwood plants (Artemisia annua) [12, 13]; their derivatives have been reported to save millions of people suffering from malaria worldwide over the decades [12]. ART can also attenuate brain inflammation and memory impairments [14]. ART could protect against oxidative stress in neurons [15, 16]. ART can easily pass through the blood-brain barrier without any obvious side effects [17].

However, since multiple underlying mechanisms are associated with hypothyroidism, other possible targets associated with ART that could ameliorate hypothyroidism and its complications were investigated such as the L1 cell adhesion molecule. The L1 cell adhesion molecule is encoded by the L1CAM gene, it contains 28 exons [18] and it is a transmembrane glycoprotein primarily discovered in the nervous system. L1 mediates cell-cell adhesion, neuronal cell survival and migration, and synaptogenesis [19]. L1 can exert key roles in learning, memory and regeneration following insult [20–23]. L1 can affect protein kinase D1 phosphorylation in mouse cerebellar granule neurons [24] and the cerebral cortex in Alzheimer’s disease [25]. In addition, berberine enhances L1 expression to promote the recovery of rats after brachial plexus root avulsion [26].

Given the essential roles of ART and L1 in normal and disease conditions, the potential functional relationship between ART and L1 in hypothyroidism was investigated. The hypothesis was that ART may exert a protective role in hypothyroidism by activating L1. In the current study, it was shown that ART could ameliorate thyroid function and complications in brain, liver, kidney and heart in adult male hypothyroid rats by upregulating L1.

## Materials and methods

### Animals

8-week male Sprague–Dawley rats (200–220 g) purchased from Hunan Medical Laboratory Animal Center were housed at 22–23ºC and 45–50% relative humidity; the light/dark cycle was 12/12 h. All experimental procedures using animals were approved by the Laboratory Animal Ethics Committee of The First Affiliated Hospital of University of South China (approval no. LL20210708002).

### Groups and treatments

The propylthiouracil (PTU) rat model was constructed according to a previous study [27]. Rats were randomly divided into the control (CTRL) group (n = 10), the PTU group (n = 10), the PTU + ART group (n = 10) and the PTU + ART + L1 siRNA group (n = 10). The rats in the CTRL group drank water normally, while the rats in the other three groups received oral administration of 15 mg/kg PTU [28]. After 28 days, the rats in the CTRL and the PTU groups were intraperitoneally injected with saline once a day, while the rats in the PTU + ART and in the PTU + ART + L1 siRNA groups were intraperitoneally injected with 5 mg/kg ART with or without L1 siRNA (sense: 5’-GCA UUAGUG GCC AUC CUU UTT-3’, antisense: 3′-TTC GUA AUC ACC GGUAGG AAA-5′) [29] once a day for 2 weeks. A total of 10 rats per group were sacrificed after anesthesia using 0.5% isoflurane, and blood samples were collected after behavioral tests.

### Behavior tests

#### Tail suspension test

The tail suspension test was carried out in rats as previously described [30, 31]. In brief, rats were hanged on a hook by ~ 1 cm from the tip of the tail using adhesive tape; the distance from the floor was 50 cm. The duration of the immobility was set as 6 min. Immobility was calculated when rats were motionless or made only small, non-escape-associated movements.

#### Forced swimming test

The forced swimming test was carried out in rats as previously described [32]. Rats were placed in a plastic cylinder with a height of 40 cm and a diameter of 20 cm filled with 15 cm of water at 25 ± 1ºC to swim. Two sessions were carried out as part of this experiment. In the first session, every rat was forced to swim for 15 min in the water-filled plastic cylinder for adapting. After 24 h, a 5-min test session was carried out. Escape responses, including climbing time and swimming time, were calculated.

#### Elevated plus maze test

The elevated plus maze test was carried out in rats as previously described [33, 34]. A total of two opposite closed arms (50 × 10 × 40 cm) and two opposite open arms (50 × 10 cm) were connected by a central square (10 × 10 cm) to make up the apparatus which was placed 50 cm above the floor. Rats were placed in the central zone facing one of the open arms during a 5-min experimental period. The percentage of open-arm entries (open/total entries x 100%) and the percentage of time spent in the open arms (open/total time spent x 100%) were determined. The more increased the open arm activity, the more serious was the anxiety behavior considered [35].

#### Morris water maze test

The Morris water maze test was carried out in rats as previously described [36]. The maze had a diameter of 1.6 m and a height of 50 cm. The pool water with a depth of 30 cm and at 22–23ºC was dyed with black food coloring. Two sessions were carried out as part of this experiment. In the first positioning navigation experiment, the rats received training for 4 days, 4 times each day. In the second experiment, rats were placed on the underwater platform to adapt for 30 s before entering the water. The swimming distance of the rat from the four quadrants and different entry points to the platform was recorded within 1 min.

On day 5, the space search experiment was conducted; after removing the platform, rats were placed into the water from the entry point in the opposite quadrant of the platform, facing the wall of the pool, and the swimming trajectory of the rats within 1 min was recorded.

#### Novel object recognition (NOR) test

The dimensions of the open-field box that was used in this experiment were 50 × 40 × 30 cm. To avoid being pushed or bitten by rats, the recognized objects were cylinders and tubes with a certain weight as well as hardness. The experiment was divided into three time periods: (i) The adaptation period; (ii) the training period; and (iii) the testing period. The discrimination index was calculated to reflect the cognitive function of the rats, which was defined by the following equation: Discrimination index = [(time spent on familiar object - time spent on novel object)/(time spent on familiar object + time spent on novel object)] x 100.

#### Y-maze test

The Y-maze test was commonly carried out on rats as described [37]. The experimental apparatus consisted of three identical labeled arms (A, B and C) at equal angles. The number and sequence for the rats to entry into each arm were recorded, as the correct sequence were BAC, ABC, or ACB. The alternation performance was calculated to evaluate the spatial differentiation memory of the rats.

### Body weight gain and food consumption

The body weight gain was calculated for each group by subtracting the initial weight (at the beginning of the experiment) from the final weight (at the end of experiment). And the food consumption per day was calculated.

### Core temperature

A non-invasive, continuous, longitudinal monitoring of core temperature in the physiological context was carried out [38, 39]. In brief, Thermochroni Button, DS1922L-F5# with a precision of 0.0625ºC was programmed to start recording Tb 1 week after implantation at 30- or 60-minute intervals and then coated with a thin layer of paraffin wax for waterproofing (3:1 ratio). The iButton was implanted surgically into the peritoneal cavity under general anesthesia using 3–5% isoflurane.

### Serum glucose determination

Blood samples were collected from the vein and then centrifuged at 1500 ×g for 30 min to obtain serum. The glucose level was determined directly after blood sampling using a kit purchased from Spectrum Diagnostics according to the method of Trinder [40].

### Serum biochemical parameter assays

Blood samples were collected from the vein and then centrifuged at 1500 × g for 30 min to obtain serum.We used enzyme-linked immunosorbent assay kits (ELISA) to estimate the levels in the serum, thyroid stimulating hormone (TSH), total triiodothyronine (T3), and total thyroxine (T4), ghrelin, glucagon-like peptide-1 (GLP-1), alanine aminotransferase (ALT), aspartateaminotransferase (AST), alkaline phosphatase (ALP), urea, creatinine, lactate dehydrogenase (LDH), creatine Kinase MB (CK-MB).

### Serum oxidative stress measurements

The levels of NO, malondialdehyde (MDA), 3-nitrotyrosine (3-NT), 4-hydroxynonenal (4-HNE), 8-oxo-deoxyguanosine (8-OHDG), catalase (CAT), glutathione (GSH), and superoxide dismutase (SOD) were determined using commercial kits according to the manufacturer’s protocols.

### Statistical analysis

Data are expressed as the mean ± standard deviation (SD). Statistical analyses were performed using GraphPad Prism 6 software with one-way analysis of variance (ANOVA) followed by a post-hoc Bonferroni test.

## Results

### ART ameliorated thyroid function by upregulating L1 in adult male hypothyroid rats

To determine the effect of ART on the thyroid function after hypothyroidism in rats, body weight, food intake per day, core body temperature, glucose level, serum thyroid stimulating hormone (TSH), tri-iodothyronine (T3) and L-thyroxine (T4) levels, and ghrelin and glucagon-like peptide-1 (GLP-1) secretion were calculated.

**Fig. 1:**
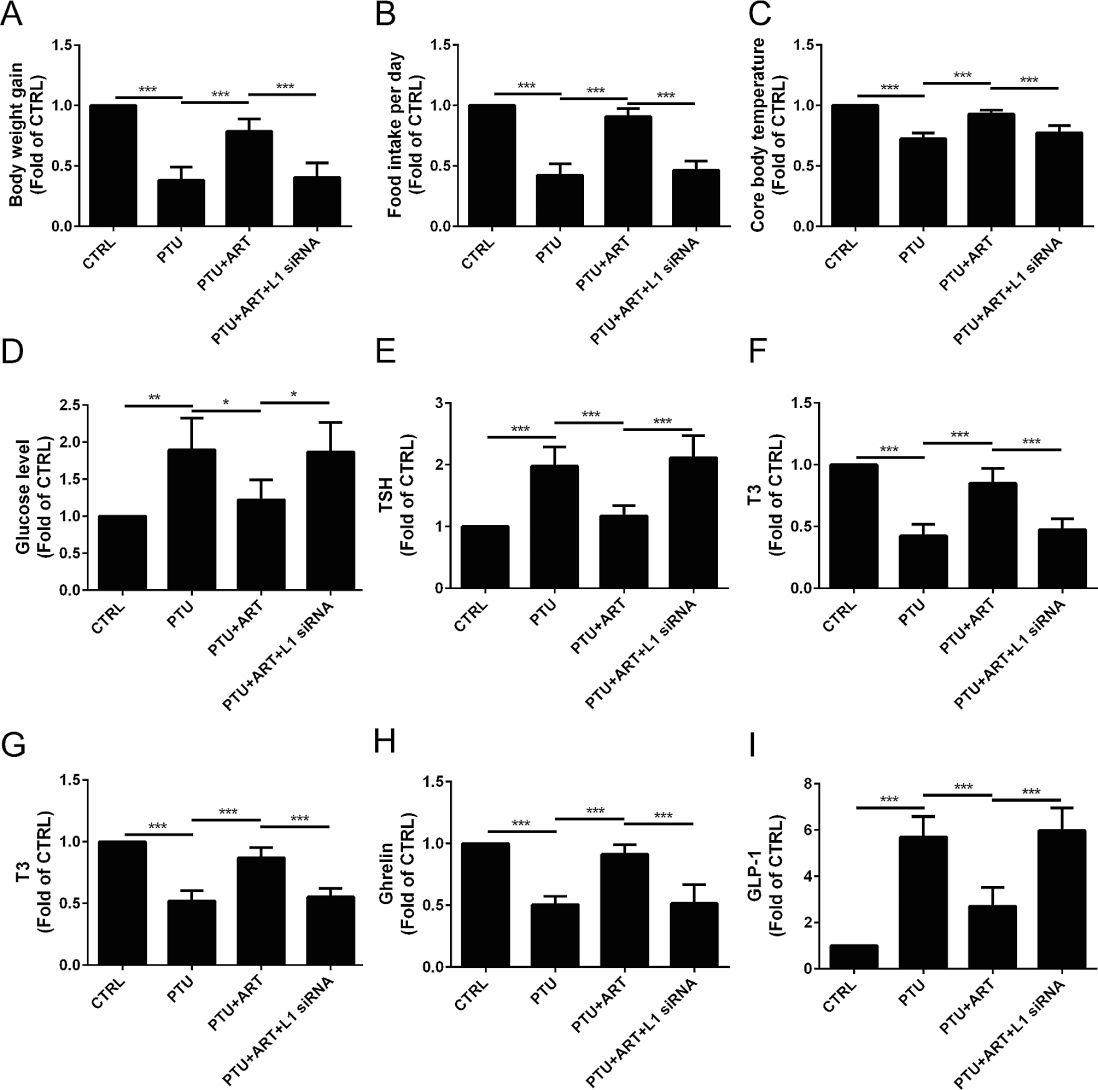
Effect of ART on the thyroid function after hypothyroidism in rats. The body weight gain (**A**), food intake per day (**B**), core body temperature (**C**) were increased, and the glucose level (**D**) was decreased by the treatment of ART. The TSH (**E**) was decreased, and T3 (**F**), T4 (**G**) were increased by the treatment of ART. Serum ghrelin (**H**) was increased, GLP-1 (**I**) was decreased by the treatment of ART. (*n* = 4) ****P < 0.0001, ***P < 0.001,**P < 0.01, *P < 0.05

Compared with the CTRL group, the body weight was increased in response to the PTU treatment, but it was decreased by ART treatment; however, following L1 inhibition using L1 siRNA, ART did not affect rat body weight (Fig. 1A). A similar pattern for food intake per day (Fig. 1B) and core body temperature (Fig. 1C), and a reverse pattern for glucose level (Fig. 1D) were observed.

Compared with the CTRL group, the TSH level was increased in response to the PTU treatment, but it was decreased by ART treatment; however, following L1 inhibition by L1 siRNA, ART did not alter the TSH level (Fig. 1E). A reverse pattern for the T3 level (Fig. 1F), and a similar pattern for the T4 level (Fig. 1G) were observed.

Compared with the CTRL group, ghrelin secretion was decreased in response to the PTU treatment, but it was increased by ART treatment; however, following L1 inhibition by L1 siRNA, ART did not alter ghrelin secretion (Fig. 1H). A reverse pattern for GLP-1secretion (Fig. 1I) was observed.

### ART mitigated depression and anxiety symptoms by upregulating L1 in adult male hypothyroid rats

To determine the effect of ART on the depression and anxiety dysfunctions after hypothyroidism in rats, a tail suspension test, a forced swimming test and an elevated plus maze test were performed.

**Fig. 2:**
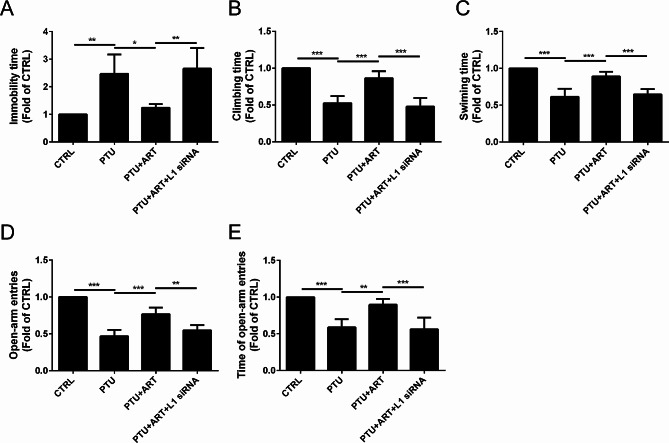
Effect of ART on the depression and anxiety symptoms after hypothyroidism in rats. The immobility time (**A**) was decreased by the treatment of ART in tail suspension test.The climbing time (**B**), swimming time (**C**) were increased by the treatment of ART in forced swimming test. The open-arm entries (**D**), the time of open-arm entries (**E**) were increased by the treatment of ART in elevated plus maze test. (*n* = 8) ****P < 0.0001, ***P < 0.001, **P < 0.01, *P < 0.05

In the tail suspension test, compared with the CTRL group, the immobility time was increased in response to the PTU treatment, but it was decreased by ART treatment; however, following L1 inhibition by L1 siRNA, ART did not alter the immobility time (Fig. 2A).

In the forced swimming test, compared with the CTRL group, the climbing time was decreased in response to the PTU treatment, but it was increased by ART treatment; however, following L1 inhibition by L1 siRNA, ART did not alter the climbing time (Fig. 2B). A similar pattern was observed for swimming time (Fig. 2C).

In the elevated plus maze test, compared with the CTRL group, the percentage of open-arm entries was decreased in response to the PTU treatment, but it was increased by ART treatment; however, following L1 inhibition by L1 siRNA, ART did not alter the percentage of open-arm entries (Fig. 2D). A similar pattern was observed for the time spent in the open arms (Fig. 2E).

### ART attenuated cognition impairments by upregulating L1 in adult male hypothyroid rats

To determine the effect of ART on the cognition dysfunction after hypothyroidism in rats, the Morris water maze test, the NOR test and the Y-maze test were performed.

**Fig. 3:**
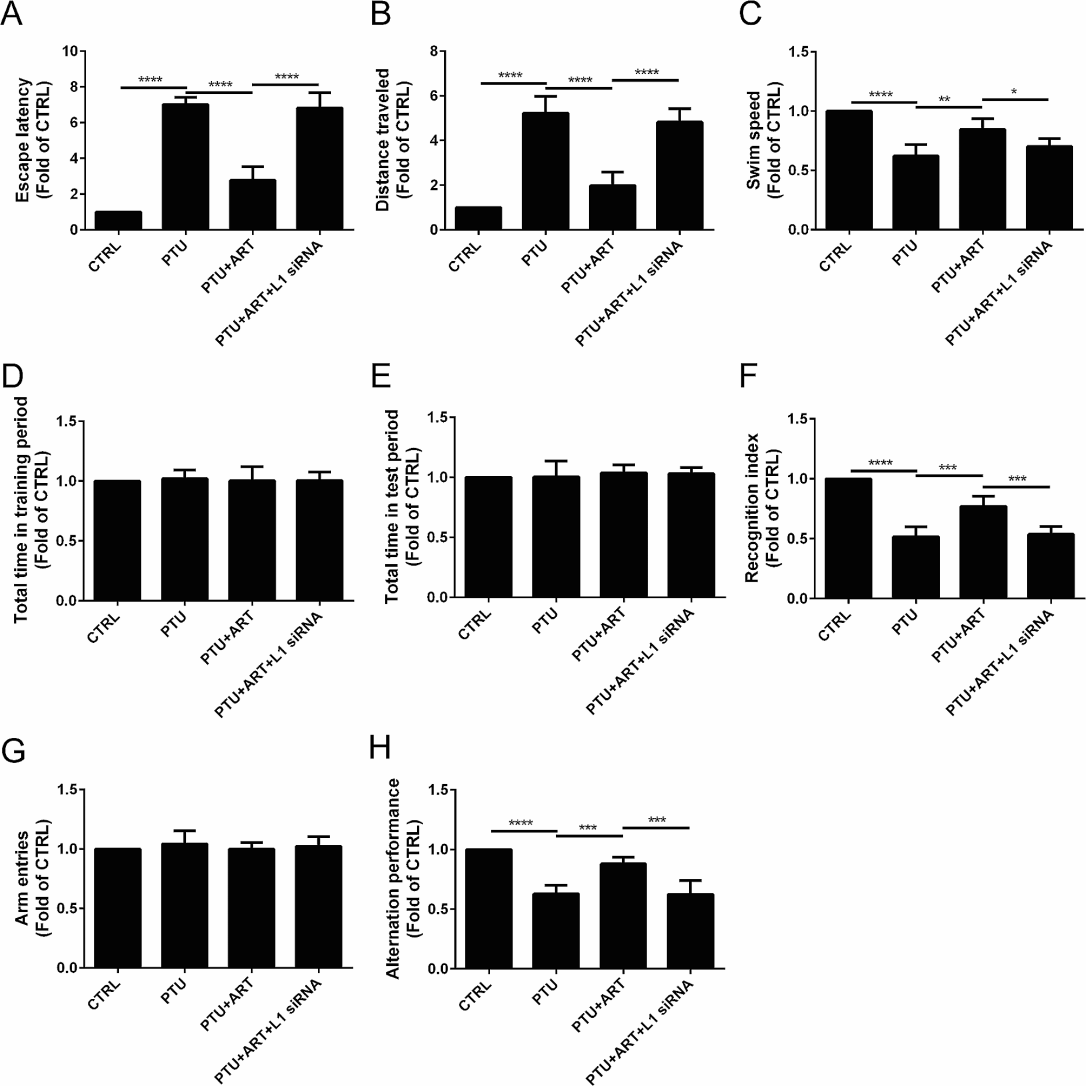
Effect of ART on the cognition impairments after hypothyroidism in rats. The escape latency (**A**) was decreased, the distance travel (**B**), and swim speed (**C**)were increased by the treatment of ART in Morris water maze test. The total time in training period (**D**), and total time in test period (**E**) were not altered, and the recognition index (**F**) was increased by the treatment of ART in NOR test. The arm entries (**G**) was not altered, the alteration performance (**H**) was increased by the treatment of ART in Y-maze test. (*n* = 8) ****P < 0.0001, ***P < 0.001, **P < 0.01, *P < 0.05

In the Morris water maze test, compared with the CTRL group, escape latency was increased in response to the PTU treatment, but it was decreased by ART treatment; however, following L1 inhibition by L1 siRNA, ART did not alter the escape latency (Fig. 3A). A similar pattern was observed for distance traveled (Fig. 3B), and a reverse pattern was observed for swim speed (Fig. 3C).

In the NOR test, no significant difference was observed in total time in both training (Fig. 3D) and test period (Fig. 3E) among the experimental groups; compared with the CTRL group, the recognition index was decreased in response to the PTU treatment, but it was increased by ART treatment; however, following L1 inhibition by L1 siRNA, ART did not alter the recognition index (Fig. 3F).

In the Y-Maze test, no significant difference was observed in the number of total arm entries in the experimental groups (Fig. 3G). Compared with the CTRL group, the alternation performance was decreased in response to the PTU treatment, but it was increased by ART treatment; however, following L1 inhibition by L1 siRNA, ART did not alter the alternation performance (Fig. 3H).

### ART improved the liver, kidney and heart functions by upregulating L1 in adult male hypothyroid rats

To determine the effect of ART on the liver, kidney and heart functions after hypothyroidism in rats, the serum alanine aminotransferase (ALT), aspartate aminotransferase (AST), alkaline phosphatase (ALP), urea, creatinine, lactate dehydrogenase (LDH), creatine kinase MB (CK-MB) activities were determined.

**Fig. 4:**
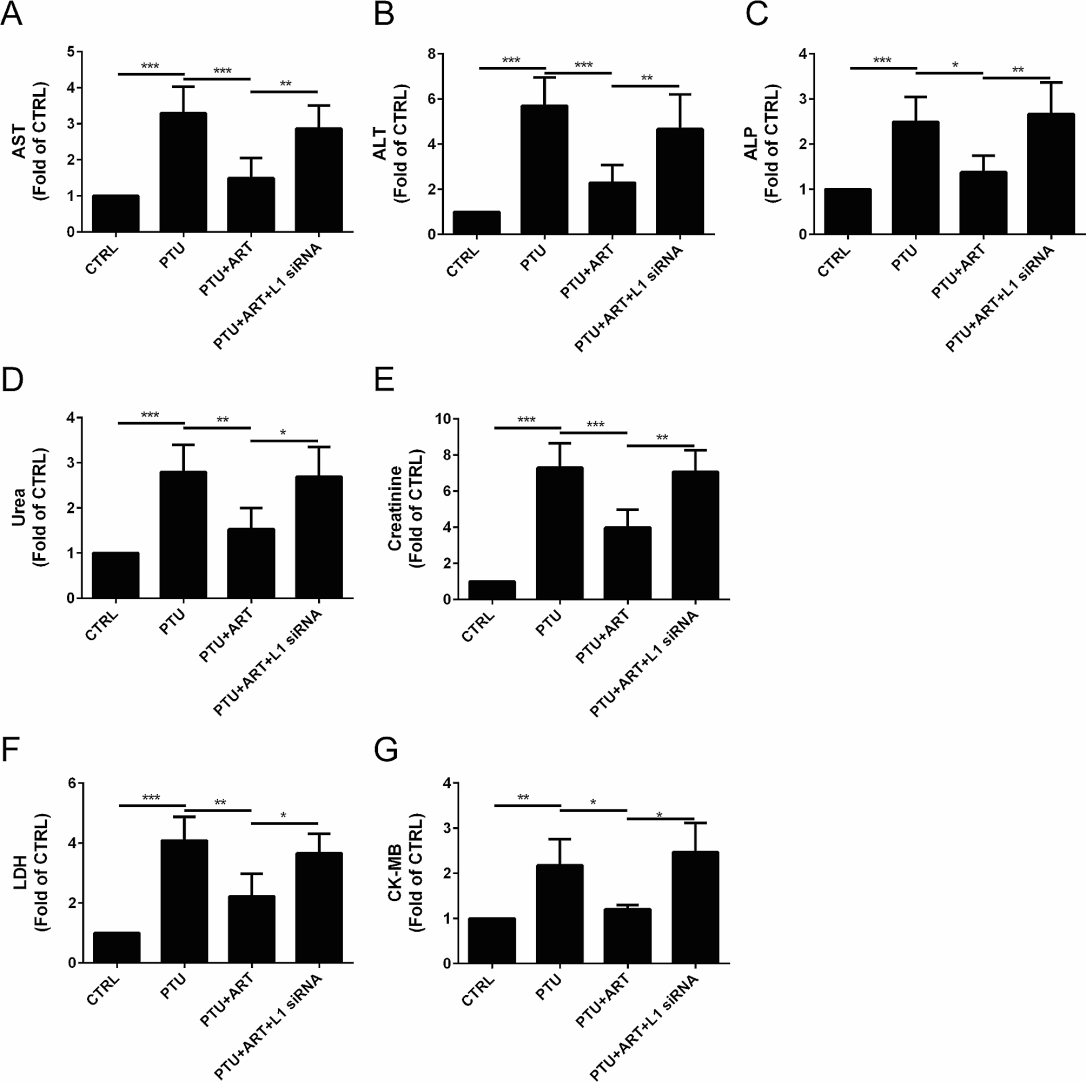
Effect of ART on the liver, kidney and heart functions after hypothyroidism in rats. ART can improve the liver function, indicated by the down-regulated AST (**A**), ALT (**B**), and ALP (**C**). ART can improve the kidney function, indicated by the down-regulated Urea (**D**), and creatinine (**E**). ART can improve the heart function, indicated by the down-regulated LDH (**F**), and CK-MB (**G**). (*n* = 4) ****P < 0.0001, ***P < 0.001, **P < 0.01, *P < 0.05

For liver function, compared with the CTRL group, the AST level was increased in response to the PTU treatment, but it was decreased by ART treatment; however, following L1 inhibition by L1 siRNA, ART did not alter the AST level (Fig. 4A). A similar pattern was observed for ALT (Fig. 4B) and ALP levels (Fig. 4C).

For kidney function, compared with the CTRL group, the urea level was increased in response to the PTU treatment, but it was decreased by ART treatment; however, following L1 inhibition by L1 siRNA, ART did not alter the urea level (Fig. 4A). A similar pattern was observed for the creatinine level (Fig. 4B).

For heart function, compared with the CTRL group, the LDH level was increased in response to the PTU treatment, but it was decreased by ART treatment; however, following L1 inhibition by L1 siRNA, ART did not alter the LDH level (Fig. 4A). A similar pattern was observed for the CK-MB level (Fig. 4B).

### ART inhibited oxidative stress by upregulating L1 in adult male hypothyroid rats

To determine the effect of ART on oxidative stress after hypothyroidism in rats, ELISA was performed to calculate the levels of NO, MDA, 3-NT, 4-HNE, 8-OHGD, CAT, GSH, and SOD in serum.

**Fig. 5:**
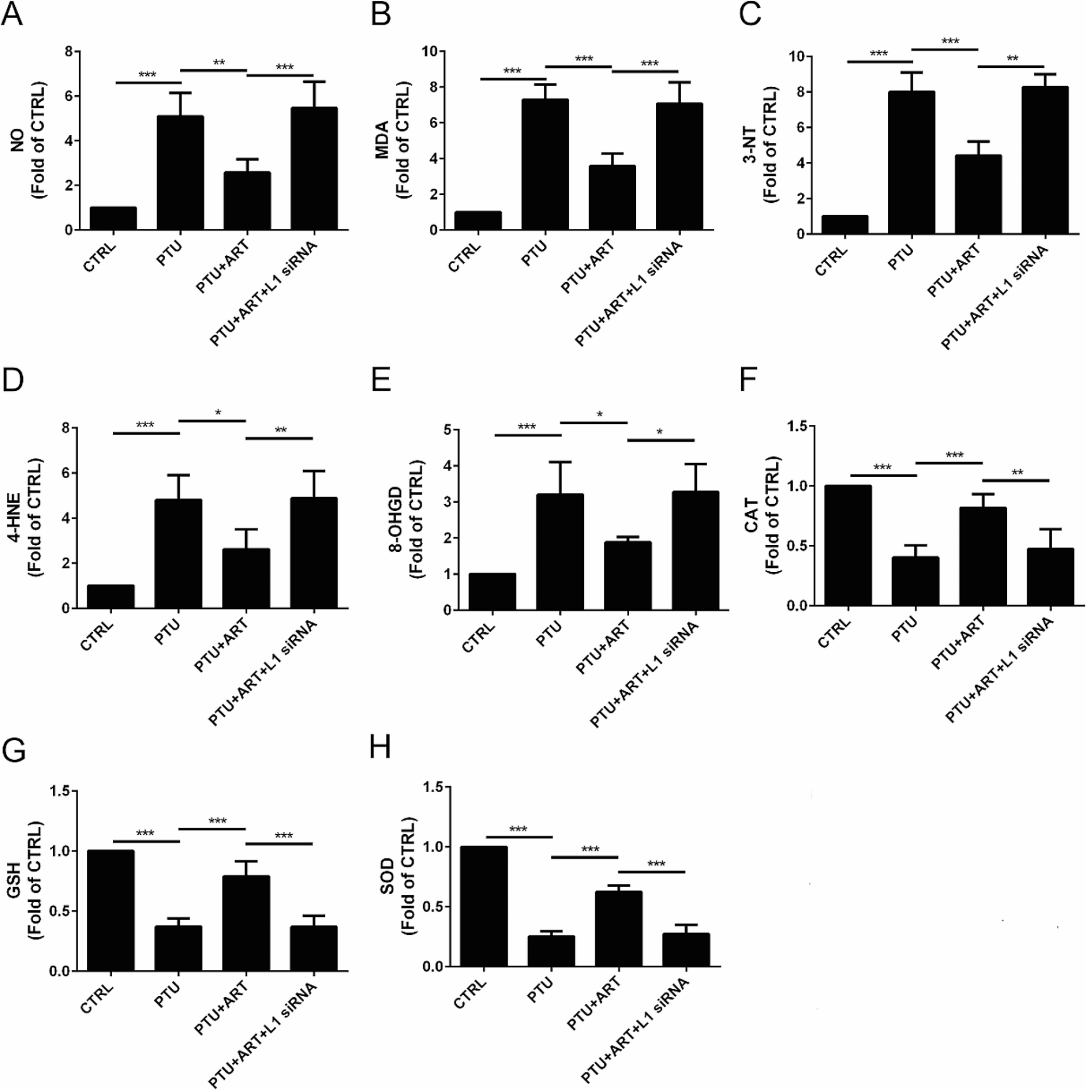
Effect of ART on the oxidative stress after hypothyroidism in rats. ART can down-regulate NO (**A**), MDA (**B**), 3-NT (**C**),4-HNE (**D**), 8-OHGD (**E**), and up-regulate CAT (**F**), GSH (**G**), SOD (**H**). (*n* = 4) ****P < 0.0001, ***P < 0.001, **P < 0.01, *P < 0.05

Compared with the CTRL group, the NO level was upregulated in response to PTU treatment, but it was down-regulated by ART treatment; however, following L1 inhibition by L1 siRNA, ART did not alter the NO level (Fig. 5A). A similar pattern was observed for MDA, 3-NT, 4-HNE and 8-OHGD (Fig. 5B-E), and a reverse pattern was observed for CAT, GSH, and SOD (Fig. 5F-H).

## Discussion

A previous study demonstrated that ART can upregulate L1 to reduce the neurological deficits in mice after intracerebral hemorrhage [41]. In the current study, it was revealed that ART can ameliorate thyroid function and complications, including depression, anxiety and cognition impairments, and associated kidney, liver and heart dysfunctions in adult male hypothyroid rats.

Hypothyroidism is characterized by a high level of TSH, and insufficient production of T3 and T4 in the serum [42]. Thyroid hormones can regulate basal energy consumption either indirectly by changing other regulatory hormones or directly by affecting carbohydrate, protein and lipid metabolism [43]. Therefore, thyroid hormone deficiency has been shown to reduce glucose availability and alter the correct absorption of glucose [44]. PTU can affect the ghrelin, an orexigenic hormone, levels in the serum, and promote GLP-1, an anorexigenic hormone, secretion, leading to decreased food intake [45]. In the current study, it was revealed that ART can ameliorate thyroid function by upregulating L1.

Thyroid hormones exert a key role in the development of brain and make contributions to maintain brain health in adulthood [46]. Also, thyroid hormones were reported to be associated with cognition [47]. It has been shown that attention, memory and spatial ability in patients suffering from hypothyroidism are notably decreased, while depression and anxiety dysfunctions were increased [48]. In the current study, it was shown that ART can mitigate depression and anxiety symptoms, and cognition impairment by upregulating L1.

The deranged lipid metabolism caused by hypothyroidism is associated with nonalcoholic fatty liver [49]. A positive relationship between aspartate AST and TSH activities has been observed in patients suffering from liver cirrhosis [50]. Also, hypothyroidism may lead to kidney function impairment [51]. A positive relationship exists between upregulated LDH and CK-MB levels in ischemic myocardial injury [52]. In the current study, it was revealed that ART can improve liver, kidney and heart functions by upregulating L1.

Oxidative stress was reported to exert an essential role in the development and pathology of thyroid diseases [53]. Compared with healthy controls, higher oxidative stress was observed in patients with hypothyroidism [54]. A previous study reported that thymoquinone can protect the testes of hypothyroid rats by suppressing oxidative stress [55]. In the current study, it was revealed that ART can inhibit oxidative stress by upregulating L1.

Taken together, the combined data indicated that ART treatment can not only ameliorate hypothyroidism, but also prevent the complications induced by hypothyroidism such as depression, anxiety and cognition impairments, and the associated kidney, liver and heart dysfunctions by increasing L1 to inhibit oxidative stress, suggesting that ART may be a novel strategy for the treatment of hypothyroidism.

## Data Availability

No datasets were generated or analysed during the current study.
